# Knowledge, attitude and behaviour on salt intake and its association with hypertension in the Malaysian population: findings from MyCoSS (Malaysian Community Salt Survey)

**DOI:** 10.1186/s41043-021-00235-0

**Published:** 2021-05-31

**Authors:** Azli Baharudin, Rashidah Ambak, Fatimah Othman, Viola Michael, Siew Man Cheong, Nor Azian Mohd. Zaki, Nur Shahida Abdul Aziz, Syafinaz Mohd. Sallehuddin, Shubash Shander Ganapathy, Lalitha Palaniveloo, Feng J. He

**Affiliations:** 1 Institute for Public Health, National Institutes of Health, Ministry of Health Malaysia, Shah Alam, Selangor Malaysia; 2 Non-Communicable Disease Section, Disease Control Division, Ministry of Health Malaysia, Putrajaya, Malaysia; 3 Sarawak General Hospital, Ministry of Health Malaysia, Kuching, Sarawak Malaysia; 4 Wolfson Institute of Preventive Medicine, London School of Medicine & Dentistry, Queen Mary University of London, London, UK

**Keywords:** Hypertension, Knowledge, Attitudes, Behaviour

## Abstract

**Background:**

High blood pressure or hypertension has become one of the main health problems, worldwide. A number of studies have proven that an increased intake of salt was related to an increased prevalence of cardiovascular diseases. Of late, its relationship with high salt intake has received a lot of attention. Studies in Malaysia have shown both rising hypertension over time as well as high salt consumption. Actions to reduce salt intake are essential to reduce hypertension and its disease burden. As such, we carried out a study to determine associations between knowledge, attitude and behaviour towards salt intake and hypertension among the Malaysian population.

**Methods:**

Data obtained from the Malaysian Community Salt Survey (MyCoSS) was used partially for this study. The survey used a cross-sectional two-stage sampling design to select a nationally representative sample of Malaysian adults aged 18 years and above living in non-institutional living quarters (LQ). Face-to-face interviews were done by trained research assistants (RA) to obtain information on sociodemography, medical report, as well as knowledge, attitude and behaviour of the respondents towards salt intake and blood pressure.

**Results:**

Majority of the respondents have been diagnosed with hypertension (61.4%) as well as knowledge of the effects of high salt intake on blood pressure (58.8%). More than half of the respondents (53.3%) said they controlled their salt intake on a regular basis. Those who knew that a high salt diet could contribute to a serious health problem (OR=0.23) as well as those who controlled their salt intake (OR=0.44) were significantly less likely to have hypertension.

**Conclusion:**

Awareness of the effects of sodium on human health, as well as the behaviour of controlling salt intake, is essential towards lowering the prevalence of hypertension among Malaysians.

## Background

Hypertension is a common condition, which if not detected and treated early, can lead to myocardial infarction, stroke, renal failure, and premature death [1]. With over 9.4 million deaths worldwide, it has been predicted that there will be up to 75% increment of global cardiovascular disease burden by the year 2020 [2–4]. The relationship between cardiovascular diseases and elevation in blood pressure in relation to salt is currently a major focus of scientific research [5]. According to Parmar et al. [6], the most important factor causing elevation of human blood pressure is salt intake. Consuming excessive dietary salt contributes to high blood pressure [7, 8]. Zhang et al. [9] suggest that the best way to control hypertension is by increasing awareness of this disease and promoting healthy salt intake behaviour.

In Malaysia, a few studies have reported that the average salt intake exceeds that recommended for health by the World Health Organization (WHO) [10, 11]. It is recommended to take not more than 5 g of salt every day in order to reduce the number of deaths related to hypertension, cardiovascular diseases as well as stroke [12]. In view of efforts to reduce salt consumption, it is imperative to find out the level of awareness among Malaysians on this issue. More importantly, we also need to investigate if awareness has any association with diagnosis of hypertension. Thus, the objective of this study was to determine knowledge, attitude, and behaviour related to salt intake, and their associations with hypertension in the Malaysian population. This information is important as a baseline for monitoring purposes and also for developing new strategies and promotional activities on salt reduction in the country.

## Methods

**Fig. 1:**
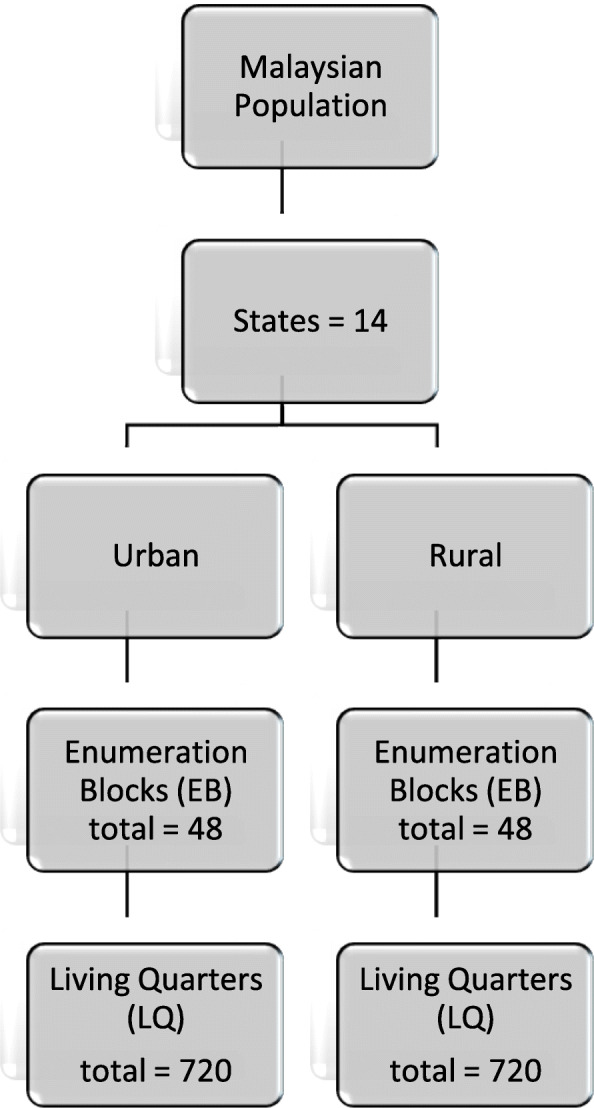
Sampling method for MyCoSS study

**Fig. 2:**
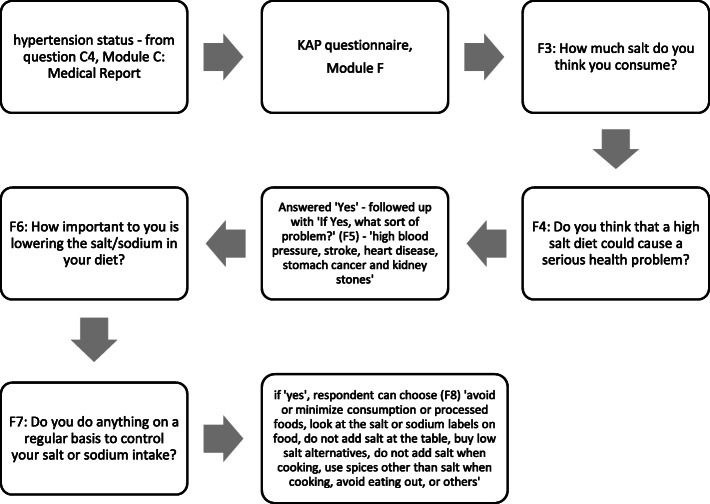
Flow chart of answering the knowledge, attitude and behaviour questions based on MyCoSS questionnaires

The data for this study was drawn from the Malaysian Community Salt Survey (MyCoSS), a nationwide cross-sectional study, conducted among Malaysian adults aged 18 years and above living in non-institutional living quarters (LQ). The sample size was calculated by assessing population prevalence based on the most recent salt study in Malaysia [13]. It was ensured to cover both urban and rural areas as well as proportionate to the size of the population according to states in Malaysia (Fig. 1). The estimated sample size was 1440 participants. Exclusion criteria of this study were those who were pregnant, recently began diuretic therapy (<4 weeks), having menses and diagnosed with kidney disease, heart failure disease or liver disease. Diagnosis of hypertension was based on medical report questions in Module C of the questionnaire (Fig. 2).

### Knowledge, attitude and behaviour

The knowledge, attitude and behaviour on salt intake questionnaire which was translated into Malay language and back-translated to English to ensure the quality of the translation, was adapted from the World Health Organization/Pan American Health Organization protocol for population-level sodium determination [14]. The flow chart in Fig. 2 shows the respondent feedback on the knowledge, attitude and behaviour towards dietary sodium intake. The data collection was carried out by trained interviewers via face-to-face interview. All interviewers were trained at the central level.

### Sociodemographic information

Interviewer-administered questionnaires were used to collect information on sociodemography and medical report of the respondents. Variables such as gender, age, occupation, ethnicity, as well as individual income were selected for use in this study.

### Statistical analysis

Statistical analysis was carried out using STATA Ver 15.0. The complex sample design of the study and weights were taken into account by using the *svy* suite for all analysis. Variance estimation was carried out using the Taylor series linearization method, and bivariate analysis was carried out using the Rao-Scott F test. Subpopulation analysis was carried out on the groups with diagnosed hypertension. Design-adjusted Wald test was used to evaluate the significance of each variable in the logistic regression model. All analysis was described using 95% confidence intervals and statistical significance at *p*-value of less than 0.05.

## Results

The total number of respondents in MyCoSS was 1440. After excluding based on the exclusion criteria, the total number was reduced to 1047 (72.7%). Table 1 shows the age, ethnic background, marital status, education and income level of the respondents. Most of the respondents were female (59.1%), aged between 55 to 64 years old (23.3%), Malay (63.2%) and married (72.7%). The majority had at least attained secondary education (48.0%), and 21.8% had higher education. Most of the respondents were housewives (28.5%), and most reported a household income of less than RM 1000/month (31.0%), and the number of respondents with diagnosed hypertension was 339 (34.1%).

**Table 1:** Sociodemographic characteristic of respondents (*n* = 1047)

Sociodemographic characteristics	Frequency	Percentage (%)
**Gender**		
Male	428	40.9
Female	619	59.1
**Age**		
< 35	232	22.2
35–44	176	16.8
45–54	216	20.6
55–64	244	23.3
> 64	179	17.1
**Ethnicity**		
Malay	662	63.2
Chinese	116	11.1
Indians	63	6.0
Other/Bumiputera	206	19.7
**Marital status**^a^		
Never married	133	12.7
Married	760	72.7
Separated/widowed	153	14.6
**Education level**		
None	96	9.2
Primary education	220	21.0
Secondary education	503	48.0
Higher	228	21.8
**Occupation**		
Public sector	144	13.8
Private sector	173	16.5
Self-employed	232	22.2
Housewife	298	28.5
Unemployed	142	13.6
Student/others	58	5.5
**Household income (RM/month)**		
< 1000	324	31.0
1000–1999	203	19.4
2000–2999	174	16.6
3000–3999	125	11.9
> 3999	221	21.1
**Diagnosed hypertension**^b^		
Yes	339	34.1
No	654	65.9

^a^Based on 1046 respondents

^b^Based on 993 respondents

Table 2 shows associations between selected sociodemographic characteristics and diagnosed hypertension status among respondents. Compared to those who claimed to have no knowledge, a significantly higher proportion (83.0%, 95% CI 64.19, 92.99) of respondents who said yes to having knowledge that a high salt diet can cause health problems have diagnosed hypertension (*p* = 0.012). Among those who said they do not take regular measures to control their salt intake, 70.1% have had a hypertension diagnosis compared to 53.3% among those who say they do regularly control their salt intake (*p* = 0.025).

**Table 2:** Association between the sociodemographic characteristics and diagnosed hypertension among respondents

Socio-demographic characteristics	Diagnosed hypertension		
	No	Yes	***p*** value
	Prevalence % (CI 95%)	Prevalence % (CI 95%)	
**Overall**	38.6% (32.41, 45.17)	61.4% (54.83, 67.59)	
**Gender**			
Male	42.0% (31.77, 52.96)	58.0% (47.04, 68.23)	0.400
Female	35.5% (26.83, 45.21)	64.5% (54.79, 73.17)	
**Age group (year)**			
<35	65.3% (27.59, 90.32)	34.7% (9.68, 72.41)	0.671
35–44	48.1% (26.63, 70.33)	51.9% (29.67, 73.37)	
45–54	33.8% (20.21, 50.73)	66.2% (49.27, 79.79)	
55–64	38.6% (27.62, 50.80)	61.4% (49.20, 72.38)	
> 64	37.7% (26.57, 50.24)	62.3% (49.76, 73.43)	
**Ethnicity**			
Malay	39.1% (31.45, 47.27)	60.9% (52.73, 68.55)	0.593
Chinese	48.1% (25.36, 71.66)	51.9% (28.34, 74.64)	
Indians	30.0% (10.78, 60.37)	70.0% (39.63, 89.22)	
Other/Bumiputera	32.0% (24.26, 40.89)	68.0% (59.11, 75.74)	
**Marital status**			
Never married	70.0% (28.27, 93.22)	30.0% (6.78, 71.73)	0.213
Married	39.5% (32.39, 47.00)	60.5% (53.00, 67.61)	
Separated/widower	31.2% (19.54, 45.84)	68.8% (54.16, 80.46)	
**Education level**			
None	48.1% (28.41, 68.46)	51.9% (31.54, 71.59)	0.305
Primary	33.6% (24.04, 44.62)	66.5% (55.38, 75.96)	
Secondary	35.9% (26.04, 47.06)	64.1% (52.94, 73.96)	
Higher	50.6% (32.89, 68.10)	49.4% (31.90, 67.11)	
**Occupation**			
Public sector	23.8% (8.20, 52.23)	76.2% (47.77, 91.80)	0.813
Private sector	39.2% (22.91, 58.32)	60.8% (41.68, 77.09)	
Self-employed	40.0% (25.62, 56.42)	60.0% (43.58, 74.38)	
Housewife	38.5% (27.08, 51.41)	61.5% (48.59, 72.92)	
Unemployed	40.0% (23.79, 58,78)	60.0% (41.22, 76.21)	
Student/others	46.4% (23.66, 70.79)	53.6% (29.21, 76.34)	
**Household Income (RM)**			
<1000	30.7% (21.04, 42.37)	69.3% (57.63, 78.96)	0.062
1000–1999	36.0% (23.97, 50.15)	64.0% (49.85, 76.03)	
2000–2999	55.8% (36.46, 73.53)	44.2% (26.47, 63.54)	
3000–3999	50.9% (31.67, 69.88)	49.1% (30.12, 68.33)	
> 3999	30.9% (19.76, 44.72)	29.9% (23.01, 37.73)	
**Knowledge, attitude and behaviour question**			
Do you think that a high salt diet could cause a serious health problem			
**No/do not know**	**17.0% (7.02, 35.81)**	**83.0% (64.19, 92.99)**	**0.012**
Yes	41.2% (34.60, 48.04)	58.8% (51.96, 65.40)	
What sort of problem (hypertension)			
No	38.0% (32.26, 44.06)	62.0% (55.94, 67.74)	0.781
Yes	40.3% (25.62, 56.96)	59.7% (43.04, 74.38)	
How important to you is lowering the salt/sodium in your diet?			
**Not important**	**39.1% (31.36, 47.50)**	**60.9% (52.50, 68.64)**	**0.958**
Very important	38.8% (28.94, 49.61)	61.2% (50.39, 71.06)	
Do you do anything on a regular basis to control your salt or sodium intake?			
**No/do not know**	**29.9% (20.81, 40.89)**	**70.1% (59.11, 79.19)**	**0.025**
Yes	46.7% (37.97, 55.67)	53.3% (44.33, 62.03)	

Table 3 shows the odds ratio between sociodemographic characteristics and diagnosed hypertension among respondents. A lower likelihood of having diagnosed hypertension was found for respondents with knowledge of high salt diet causing a serious health problem (OR= 0.23, 95% CI 0.06, 0.85), those who practise controlling salt intake (OR= 0.44, 95% CI 0.21, 0.91) and those with household income of RM 2000 to RM 2999 (OR= 0.28, 95% CI 0.13, 0.62).

**Table 3:** Odds ratio between sociodemographic characteristics and diagnosed hypertension among respondents

Socio-demographic characteristics	OR (95% CI)	p value
**Gender**		
Male	0.40 (0.13, 1.19)	0.099
Female	1 (ref)	
**Age group (year)**		
<35	1 (ref)	
35–44	1.66 (0.07, 41.08)	0.755
45–54	3.63 (0.21, 62.89)	0.372
55–64	1.99 (0.11, 36.11)	0.639
> 64	2.75 (0.13, 58.77)	0.514
**Ethnicity**		
Malay	1 (ref)	
Chinese	0.47 (0.14, 1.63)	0.232
Indians	2.00 (0.43, 9.31)	0.372
Other/Bumiputera	1.07 (0.43, 2.66)	0.891
**Marital status**		
Never married	0.11 (0.00, 18.39)	0.400
Married	1 (ref)	
Separated/widower	1.55 (0.55, 4.40)	0.402
**Education level**		
None	1 (ref)	
Primary	2.36 (0.78, 7.19)	0.128
Secondary	2.62 (0.78, 8.86)	0.119
Higher	0.96 (0.21, 4.47)	0.956
**Occupation**		
Public sector	1 (ref)	
Private sector	0.47 (0.05, 4.65)	0.516
Self-employed	0.52 (0.10, 2.69)	0.429
Housewife	0.26 (0.05, 1.24)	0.089
Unemployed	0.48 (0.09, 2.47)	0.374
Student/others	0.47 (0.0.84, 2.58)	0.378
**Household income (RM)**		
< 1000	1 (ref)	
1000–1999	0.88 (0.35, 2.26)	0.798
2000–2999	0.28 (0.13, 0.62)	0.002
3000–3999	0.28 (0.07, 1.12)	0.071
> 3999	1.74 (0.63, 4.83)	0.280
Do you think that a high salt diet could cause a serious health		
No/do not know	1 (ref)	
Yes	0.23 (0.06, 0.85)	0.029
What sort of problem (hypertension)		
No	1 (ref)	
Yes	1.63 (0.78, 3.42)	0.195
How important to you is lowering the salt/sodium in your diet?		
Not important	1 (ref)	
Very important	1.48 (0.92, 2.75)	0.217
Do you do anything on a regular basis to control your salt or sodium intake?		
No/do not know	1 (ref)	
Yes	0.44 (0.21, 0.91)	0.028

## Discussion

Hypertension is a common and serious worldwide public health problem that can lead to high mortality and morbidity rates. It has been identified as one major risk factor for cardiovascular diseases and chronic kidney disease. This study revealed that the prevalence of hypertension was about 34.1%. The prevalence obtained in this study seems to be similar closer to 32.7% as cited by NHMS 2011 but lower than the WHO 2008 estimate (34.0%) in the South-East Asia region [15]. The difference in the WHO 2008 estimate may be due to the wider scope and population covered in the WHO study.

From several previous studies, it is known that knowledge, attitude and behaviour toward hypertension and salt intake play a significant role in controlling blood pressure and as a preventive measure for hypertension [16, 17]. In this study, majority of the respondents (83.0%) had good knowledge of salt intake as they agreed that a high salt diet could contribute to serious health problems. On the other hand, the findings also indicated that some respondents did not have basic exposure to information on hypertension and salt intake. This finding of widespread awareness coincides with several other studies which found a good level of exposure and knowledge about hypertension and salt intake in their study populations [18–21]. However, despite knowing the facts, 60.9% of respondents perceived lowering their salt intake as not at all important. This is indicative of a poor attitude. Furthermore, almost 30.0% of respondents (total from *n*=993) did not take any regular measures to control salt or sodium intake. Even more disappointing, a majority of those who did not control their salt intake have had hypertension diagnosed. Three studies in Nigeria and Bangladesh showed comparable results to ours whereby a majority of their respondents had good knowledge but poor practice related to hypertension and salt intake [22–24]. It is possible that the poor attitude in our study population on the importance of lowering salt or sodium intake may be due to lack of awareness. The MySalt 2015 study in Malaysia showed a contrasting result, as the respondents involved were health staff, that there were good knowledge and attitude towards salt intake as well as moderate to good practise on salt control or sodium intake [13].

For risk assessment, our study identified respondents’ household income and knowledge of salt intake as the lifestyle risk factor associated with hypertension. Compared to other studies that showed age as an important non-modifiable risk factor for hypertension [25–27], our study showed a contrasting result, where the age of respondents was not a significant predictor for hypertension. From the data analysis, we found that respondents with a monthly household income of RM 2000 to RM 2999 had lower odds of getting hypertension compared to respondents with a household income of less than RM 1000. Based on NHMS 2011 findings, the lower (< RM 400) and higher (RM 3000 to RM 3999) household income levels had higher odds of getting hypertension [28]. Previous studies revealed that the association between respondents’ economic status, whether in terms of wealth index or household asset, and the prevalence of hypertension was controversial [29]. This may be due to differences in study population or other confounding factors such as cultural and sociobehavioural factors.

Low awareness towards salt intake is considered as a risk factor for hypertension [21]. The knowledge that high salt consumption could cause a serious health problem is related to diagnosed hypertension. A study conducted in Nigeria demonstrated that inadequate hypertension-related knowledge was an independent risk factor for hypertension [17]. Pandit et al. [30] revealed that educational status and level of knowledge about hypertension were important to help in controlling blood pressure levels especially among patients with diagnosed hypertension. Hence, knowledge of hypertension and salt intake is very important for the general population so that they could be aware as well as evaluate their general health status [31].

Moreover, practising healthier dietary behaviour requires one to possess both knowledge and skill since the sodium intake is not fully determined by knowledge, attitude and behaviour of consumer but other roles such as cultures. Delivering education to population should not be limited to information on health and sodium but also provide both practical and culturally appropriate to improve their diet [32]. People should be made aware of hidden sodium and other sources of sodium in their diet. Furthermore, campaigns on using low sodium condiments or alternative forms of flavouring should be introduced and recommended [11].

The barriers of poor awareness, attitude and behaviours should be a concern and be given attention in hypertension preventive efforts. Educational and awareness programmes should be developed based on the demands of the society in order to improve the knowledge, change the attitude and enhance the behaviour within the general population towards hypertension and salt intake. Besides, gaps between knowledge, attitudes and behaviours should be identified as it is crucial to formulate and implement clear strategies by which the knowledge and positive attitudes can be converted into beneficial behaviours [6].

## Limitations

Knowledge, attitudes and behaviour questionnaire is based on limited questions and self-reported data. Therefore, the questions may have been misunderstood by the respondent. The tendency of having social desirability bias is also high. Moreover, question with choices lack of flexibility due to fixed choices given. This study was based on MyCoSS which was the first study in Malaysia related to salt intake, hence, not enough data to compare with.

This study is a cross-sectional study. Hence, we cannot determine the relationship between respondents’ knowledge of the amount of salt in the diet and diagnosed hypertension as a causal relationship. This study was based on MyCoSS which was the first study in Malaysia related to salt intake, hence, not enough data to compare with.

## Conclusions

In conclusion, having the knowledge of the amount of salt in the diet and the effect of salt towards human health, as well as the behaviour of regulating the intake of salt, is related to hypertension among the Malaysian population. This important information could be the baseline information towards future studies as well as in enabling steps to be taken towards reducing salt consumption among Malaysians.

## Data Availability

The datasets used and/or analysed during the current study are available from the corresponding author on reasonable requests.

## Abbreviations

MyCoSS: Malaysian Community Salt Survey
WHO: World Health Organization
CI: Confidence interval
